# Supplementary material for: Unveiling the role of interleukin-13 in liver fibrosis of chronic hepatitis B patients: Development of a predictive model
Source: PLoS One. 2026 Mar 23;21(3):e0344791. doi: 10.1371/journal.pone.0344791 (PMC13008083; doi:10.1371/journal.pone.0344791)
Supplement: S1 Table — Available at https://doi.org/10.6084/m9.figshare.31479409. (DOCX) [file pone.0344791.s001.docx]

**S1 Table. Exploratory Interaction Analyses in Multiple Linear Regression Models**

| **Interaction Term** | **Unstandardized B** | **Standard Error** | **p-value** | **Model R²** |
| --- | --- | --- | --- | --- |
| IL-13 × Age | 0.005 | 0.002 | 0.02 | 0.061 |
| IL-13 × Platelet Count | -0.01 | 0.004 | 0.022 | 0.060 |

*Interaction terms were constructed using mean-centered variables. Each interaction was evaluated in a separate multiple linear regression model
