# Supplementary material for: Unveiling the role of interleukin-13 in liver fibrosis of chronic hepatitis B patients: Development of a predictive model
Source: PLoS One. 2026 Mar 23;21(3):e0344791. doi: 10.1371/journal.pone.0344791 (PMC13008083; doi:10.1371/journal.pone.0344791)
Supplement: S4 Table — Available at https://doi.org/10.6084/m9.figshare.28425038.v1. (DOCX) [file pone.0344791.s004.docx]

Analytical Code – Model Development using IL-13

|  | **Data Collection** |  |
| --- | --- | --- |
| 1 | Collect demographic data |  |
| 2 | Collect laboratory data needed to compute predictor variable |  |
| 3 | Collect Transient Elastography result |  |
| 4 | Ensuring no missing data is available |  |
|  | **Data analyses** |  |
| 1 | Check the distribution of the data  SPSS: Analyze - Descriptive Statistics - Explore – Put Numerical data on Dependent List – Plots – Normality plots with tests |  |
| 2 | ROC to find the best cut-off for IL-13 in determining High-risk liver fibrosis group.  SPSS: Analyze – ROC Curve – Put Predictor Variable on Test Variable – Put Target Condition on State Variable – Fill the Target condition code in Value of State Variable – Check ROC, Standard Error Confidence Interval, Coordinate points  Find the Biggest Youden’s Index:  (Sensitivity + Specificity – 1) |  |
| 3 | **Outcome:**  Liver stiffness measurement (LSM) transformed to obtain a normal distribution, through compute variable command  inverseLSM =$\frac{1}{LSM+1}$  SPSS: Transform – Compute Variable – Numeric expression |  |
| 5 | **Multivariate Linear Regression and assumptions**  SPSS: Analyze – Regression – Linear – Put outcome on Dependent – Put Predictors on Independents  Statistics:  Check Estimates, CI 95%, Model Fit, R Squared Change, Descriptives, Part and partial correlations, Collinearity diagnostics, Durbin-Watson, Casewise diagnostics outliers outside 3 SD.  Plots:  Y: ZRESID  X: ZPRED  Standardized residual Plots  Save:  Unstandardized predicted values, unstandardized residuals, standardized predicted values, standardized residuals, Cook’s  **Additional Correlations test:**  Analyze – Correlate – Pearson/ Spearman  **Additional computerized Breusch pagan test:**  Compute Residual Value variable to squared residual value: Transform – Compute variable  Use Squared Residual value as the outcome in Multiple linear regression analysis with the same predictors. Look for p-value of ANOVA. p-value <0.05 means a heteroscedasticity. |  |
