# Supplementary figures and images for: Unveiling the role of interleukin-13 in liver fibrosis of chronic hepatitis B patients: Development of a predictive model
Source: PLoS One. 2026 Mar 23;21(3):e0344791. doi: 10.1371/journal.pone.0344791 (PMC13008083; doi:10.1371/journal.pone.0344791)

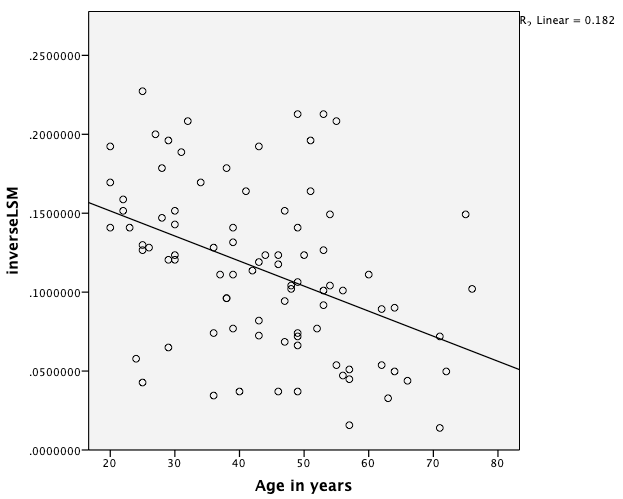


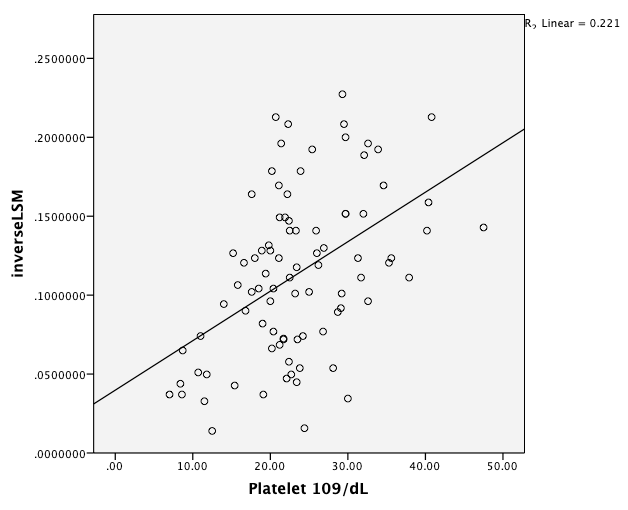


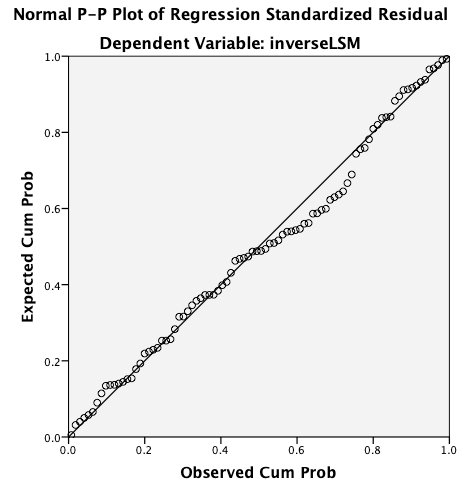


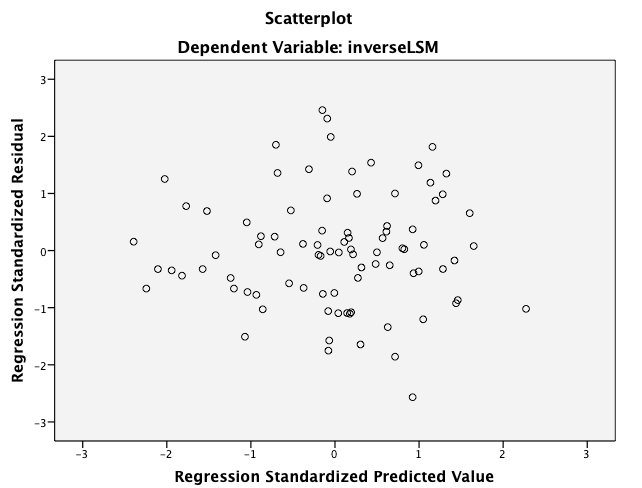


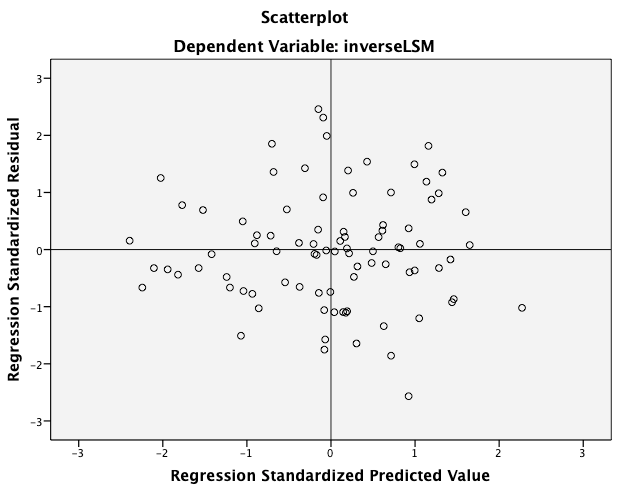

Supplement: S1 Fig — Available at https://doi.org/10.6084/m9.figshare.31479883. (DOCX) [file pone.0344791.s005.docx]
