# Supplementary material for: Unveiling the role of interleukin-13 in liver fibrosis of chronic hepatitis B patients: Development of a predictive model
Source: PLoS One. 2026 Mar 23;21(3):e0344791. doi: 10.1371/journal.pone.0344791 (PMC13008083; doi:10.1371/journal.pone.0344791)
Supplement: Attachment — Submitted filename: Response letter BB rev January fin.docx [file pone.0344791.s007.docx]

To:

Editors and Reviewers,

PLOS One

Subject: Submission of Revised Manuscript

I hope this letter finds you well. I would like to express my sincere gratitude for the feedback provided by the editor and reviewer for our manuscript entitled "Unveiling the Role of Interleukin-13 in Liver Fibrosis of Chronic Hepatitis B Patients: Development of a Predictive Model" Manuscript ID: PONE-D-25-42985R1. Your insightful comments and suggestions have significantly contributed to the enhancement of the quality and clarity of our work.

In response to the reviewers' feedback, we have carefully addressed each comment and made substantial revisions to improve the overall quality of the manuscript. Enclosed is the revised manuscript and a marked-up copy highlighting the specific changes made in response to the reviewer's suggestions. These revisions address the concerns the reviewers raised and improve the manuscript's overall quality. We value the reviewers' time and effort and believe our manuscript now meets the high standards of PLOS One.

The authors declare that they have no competing interests. Specifically, within the five years preceding the conduct of this research and the preparation of this manuscript, the authors have not had any financial relationships or activities that could be perceived as influencing the work, including but not limited to employment, consultancy, board membership, stock or share ownership, patent applications (pending or issued), research grants, travel grants, honoraria, or gifts from any commercial or non-commercial entities. The authors also declare no non-financial competing interests, including acting as expert witnesses, membership in government or advisory boards, affiliations with advocacy or lobbying organizations, relationships with funding bodies or educational companies, personal or professional relationships with individuals involved in the submission or evaluation of this manuscript, or personal convictions that could reasonably be perceived as influencing the objectivity of the research. Interests outside the five-year time frame that could reasonably be perceived as competing are also absent.

Thank you for considering our resubmission. We look forward to your feedback and the potential acceptance of our work for publication.

Sincerely,

M. Begawan Bestari, MD, MHS, PhD, FASGE, FACG

Division of Gastroenterohepatology, Department of Internal Medicine

Hasan Sadikin General Hospital – Faculty of Medicine, Universitas Padjadjaran

ORCID ID: 0000-0002-6911-8213

Bandung, Indonesia

E-mail: [begawan@unpad.ac.id](mailto:begawan@unpad.ac.id)

Suggestions from the editors and reviewers:

**Reviewer #1**

Introduction

1. Comment:

The rationale for selecting IL-13, age, and platelet count is stated; however, it is unclear why only these three factors were chosen. For example, why was ALT not considered as a potential predictor?

Response:

One of the primary objectives of this study was to specifically evaluate the independent role of interleukin-13 (IL-13) in liver fibrosis among patients with chronic hepatitis B. Therefore, we intentionally limited the model to a small number of clinically accessible variables to maintain conceptual focus on IL-13 rather than to develop a comprehensive replacement model for existing fibrosis indices. Established predictors such as ALT and AST have been extensively validated in prior studies and were not included to preserve the exploratory nature of this analysis. *The relevant revision is presented in the last paragraph of the Introduction section and further strengthened in paragraph 4 of the Discussion section.*

2. Comment:

The rationale for IL-13 should be stronger. The connection between IL-13 and chronic hepatitis B is not well established in the introduction.

Response:

We have strengthened the scientific rationale in the final paragraph of the Introduction by expanding the discussion on IL-13–mediated profibrotic mechanisms, including hepatic stellate cell activation, collagen production, and profibrotic signaling pathways, while acknowledging conflicting results from previous studies. *The relevant revision can be found in the last paragraph of the Introduction section.*

Methods

3. Comment:

Why was the IL-13 variable converted into categorical data? This may lead to loss of statistical power and information.

Response:

Multiple transformations of IL-13 values, including logarithmic and variance-stabilizing approaches, were explored but failed to achieve approximate normality or resulted in unstable effect estimates. Given evidence of non-linearity and potential threshold behavior, dichotomization using an empirically derived cut-off based on ROC analysis and the Youden index provided a more stable and clinically interpretable variable while preserving the observed association within multivariable models.

4. Comment:

The cut-off value may not be clinically meaningful or generalizable to other cohorts.

Response:

We acknowledge that the identified IL-13 cut-off may be cohort- and assay-dependent. Accordingly, we explicitly state in the manuscript that external validation in independent populations is required to assess its generalizability. *This can be found in the last paragraph of the Discussion section.*

Results

5. Comment:

The statement regarding the absence of multicollinearity is unclear because correlation data are not shown.

Response:

*Pairwise correlation coefficients between predictors are presented in the r column of Table 3 and can be found on page 6*. All correlation coefficients were below 0.7, supporting the absence of problematic multicollinearity prior to multivariable regression analysis.

6. Comment:

Consider including interaction terms, such as Age × IL-13.

Response:

Potential interaction terms, including Age × IL-13, were explored during model development. These interaction terms did not reach statistical significance and did not meaningfully improve model performance. The main effects of age, platelet count, and IL-13 remained stable across models.

Discussion

7. Comment:

The clinical relevance of the model should be discussed, including comparison with existing models such as FIB-4.

Response:

FIB-4 was not evaluated in this study because its constituent parameters were not part of the predefined variable set for model development. This study was intentionally designed to explore the independent contribution of IL-13 rather than to compare or replace established fibrosis indices. *The scope has been clarified in paragraph 4 of the Discussion section.*

8. Comment:

A deeper discussion is needed regarding discrepancies between this study and previous IL-13 studies.

Response:

We have expanded the Discussion to address potential biological, cohort-specific, and methodological explanations for differences between our findings and prior studies, including population characteristics, outcome definitions, IL-13 measurement techniques, and analytical approaches. *This explanation can be found in the last paragraph of the Discussion section.*

**Reviewer #2**

1. Comment:

The sample size (n = 88) is small for predictive modeling, and model performance may be overestimated.

Response:

We acknowledge that the sample size is suitable for exploratory model development but may be insufficient for definitive prediction modeling. *Sample size justification appears in paragraph 2 of the Participants subsection, and the need for external validation is stated in the last paragraph of the Discussion section.*

2. Comment:

The model was built and evaluated using the same dataset, limiting generalizability.

Response:

We agree and emphasize that external validation in independent cohorts is required to assess the generalizability and stability of the proposed model. *This can be found in the last paragraph of the Discussion section.*

3. Comment:

An R² of 0.37 limits clinical usefulness. Should additional variables be included?

Response:

An R² of 0.37 reflects the multifactorial nature of liver fibrosis. The model was not intended as a standalone clinical decision tool but as an exploratory analysis to evaluate the independent association of IL-13 with liver stiffness. *This point has been addressed in paragraph 4 of the Discussion section.*

4. Comment:

How was the IL-13 cut-off determined, and should it be validated?

Response:

The IL-13 cut-off was derived using ROC analysis and the Youden index, as documented in the analytical workflow. We acknowledge that this threshold requires validation in other populations and laboratory settings. *This has been addressed in the last paragraph of the Discussion section.*

5. Comment:

The mechanism of IL-13 in CHB-related fibrosis is not fully understood. Are there plans to conduct in vitro or in vivo experiments to validate its role?

Response:

Based on our clinical and scientific background, we consider external validation of our predictor model in independent cohorts to be the most appropriate next step. *(last paragraph of Discussion section)* The relevant pathophysiological mechanisms have been discussed in detail in the *Discussion section paragraph 3* with reference to existing literature

6. Comment:

Potential confounders influencing liver stiffness are not discussed in depth.

Response:

Major confounders were minimized through strict exclusion criteria, including metabolic, infectious, cardiovascular, renal, and malignant conditions. Blood sampling and transient elastography were performed prior to initiation of hepatitis B therapy. *These details have been clarified in the first paragraph of Methods section.*

Minor concern:

1. Comment:

The notation “x 10⁹/L” should be replaced with the multiplication symbol “×”.

Response:

All instances have been corrected to the standard scientific notation “× 10⁹/L” throughout the manuscript.

**Reviewer #3**

Major revision

1. Comment:

The independent role of IL-13 in CHB-related fibrosis should be better discussed in the Introduction.

Response:

The Introduction has been revised to include a more comprehensive literature summary on IL-13–mediated fibrogenesis across chronic liver diseases, thereby strengthening the rationale for its evaluation in chronic hepatitis B. *This is presented in the last paragraph of the Introduction section.*

2. Comment:

Clarify whether IL-13 remains significant after adjustment for age and platelet count.

Response:

Yes. After adjustment for age and platelet count, IL-13 remained an independent and statistically significant predictor of liver stiffness measurement, as shown in Table 4 (p = 0.002) *at the beginning of page 7.*

3. Comment:

Benchmarking against FIB-4 or APRI could be considered.

Response:

This study was designed as an exploratory analysis focused on IL-13 rather than on comparative model performance. Benchmarking against existing fibrosis indices represents an important direction for future research.

Minor revision

1. Comment:

Provide a visual representation of model performance.

Response:

We have added a supplementary file containing multiple scatterplots illustrating data distribution and model stability. Model robustness has also been demonstrated through several diagnostic analyses presented in the manuscript, including the Durbin–Watson test, plots of observed versus standardized predicted values, standardized residual scatterplots, computerized Breusch–Pagan analysis, Cook’s distance and standardized residual evaluation, P–P plots, and tolerance analysis.

***We believe that all reviewer and editorial comments have now been fully addressed***
