# Supplementary material for: Unveiling the role of interleukin-13 in liver fibrosis of chronic hepatitis B patients: Development of a predictive model
Source: PLoS One. 2026 Mar 23;21(3):e0344791. doi: 10.1371/journal.pone.0344791 (PMC13008083; doi:10.1371/journal.pone.0344791)
Supplement: Attachment — Submitted filename: Response letter IL-13 - Feb .docx [file pone.0344791.s008.docx]

To:

Editors and Reviewers,

PLOS One

Subject: Submission of Revised Manuscript

I hope this letter finds you well. I would like to express my sincere gratitude for the feedback provided by the editor and reviewer for our manuscript entitled "Unveiling the Role of Interleukin-13 in Liver Fibrosis of Chronic Hepatitis B Patients: Development of a Predictive Model" Manuscript ID: PONE-D-25-42985R1. Your insightful comments and suggestions have significantly contributed to the enhancement of the quality and clarity of our work.

In response to the reviewers' feedback, we have carefully addressed each comment and made substantial revisions to improve the overall quality of the manuscript. Enclosed is the revised manuscript and a marked-up copy highlighting the specific changes made in response to the reviewer's suggestions. These revisions address the concerns the reviewers raised and improve the manuscript's overall quality. We value the reviewers' time and effort and believe our manuscript now meets the high standards of PLOS One.

The authors declare that they have no competing interests. Specifically, within the five years preceding the conduct of this research and the preparation of this manuscript, the authors have not had any financial relationships or activities that could be perceived as influencing the work, including but not limited to employment, consultancy, board membership, stock or share ownership, patent applications (pending or issued), research grants, travel grants, honoraria, or gifts from any commercial or non-commercial entities. The authors also declare no non-financial competing interests, including acting as expert witnesses, membership in government or advisory boards, affiliations with advocacy or lobbying organizations, relationships with funding bodies or educational companies, personal or professional relationships with individuals involved in the submission or evaluation of this manuscript, or personal convictions that could reasonably be perceived as influencing the objectivity of the research. Interests outside the five-year time frame that could reasonably be perceived as competing are also absent.

Thank you for considering our revision. We look forward to your feedback and the potential acceptance of our work for publication.

Sincerely,

M. Begawan Bestari, MD, MHS, PhD, FASGE, FACG

Division of Gastroenterohepatology, Department of Internal Medicine

Hasan Sadikin General Hospital – Faculty of Medicine, Universitas Padjadjaran

ORCID ID: 0000-0002-6911-8213

Bandung, Indonesia

E-mail: [begawan@unpad.ac.id](mailto:begawan@unpad.ac.id)

Suggestions from the editors and reviewers:

**Reviewer #1**

1. Comment:

Dichotomization may reduce statistical power and produce cohort-specific cut-offs. Additional evidence of robustness (e.g., internal validation or sensitivity analysis) is suggested, and the limitation regarding cohort- and assay-dependence should be more clearly stated.

Response:

We agree that dichotomization of a continuous variable may reduce statistical power and yield cohort-specific thresholds. Accordingly, we have strengthened the Limitations section to explicitly state that the IL-13 cut-off was derived from the internal cohort and may be influenced by population characteristics and assay differences. We also clarify that dichotomization may lead to population-specific thresholds and that the identified cut-off should be interpreted with caution. Furthermore, we emphasize the need for validation in larger and independent populations to assess the robustness and generalizability of both the IL-13 cut-off and the predictive model. *The relevant revisions are presented in the third paragraph of the Discussion section, while the study limitations are further detailed in the sixth paragraph of the Discussion section.*

2. Comment:

Although the sample size meets minimum criteria for model development, the reviewer recommends more clearly acknowledging that a relatively small cohort may affect model stability and reduce the ability to detect interaction effects.

Response:

We appreciate this important point. In the revised manuscript, we have explicitly acknowledged that, despite meeting minimum sample size requirements, the modest number of participants may limit model stability and reduce the ability to detect interaction effects. This limitation has been clearly stated in the Limitations section to ensure transparent interpretation of the findings. *The relevant revision has been clearly stated in the sixth paragraph of the Discussion section.*

3. Comment:

Include a supplementary table of the interaction model results to improve transparency, particularly given the limited power to detect interactions.

Response:

We agree that reporting the interaction model results enhances transparency. Accordingly, we have included a supplementary table presenting the results of the interaction analyses. In exploratory analyses, interactions between IL-13 and age (p = 0.02) and between IL-13 and platelet count (p = 0.022) were observed. However, these models explained only a limited proportion of the variance (R² ≈ 0.06). These findings support our decision to exclude interaction terms from the final model due to their limited informative contribution. *The interaction analyses are reported as exploratory findings and are presented in Supplementary Table 1, summarized in the final paragraph of the Model Development subsection, and discussed in the fourth paragraph of the Discussion section.*

**Reviewer #2**

1. Comment:

Consider incorporating the two minor suggestions (strengthening the limitation on external validation in the text and adding a brief phrase on the exploratory nature of the IL-13 cut-off in Results) before final publication.

Response:

We thank the reviewer for these helpful suggestions. In response, we clarified the need for external validation by strengthening the Limitations section and emphasizing validation in larger and independent populations. We also added a brief explanation in the Discussion section to clarify the rationale behind the derived IL-13 cut-off. *The study limitations are discussed in more detail in the sixth paragraph of the Discussion section, while the use of the Youden index is explained in the third paragraph of the Discussion section.*

**Reviewer #3**

1. Comment:

No further comments.

Response:

We sincerely thank the reviewer for the positive evaluation of our manuscript and for confirming its suitability for publication.

***We believe that all reviewer and editorial comments have now been fully addressed***
